# Comparison of three progesterone quantification methods using blood samples drawn from bitches during the periovulatory phase

**DOI:** 10.14202/vetworld.2022.119-123

**Published:** 2022-01-24

**Authors:** Hassan A. Hussein, Gerhard Schuler, Theresa Conze, Axel Wehrend

**Affiliations:** 1 Department of Theriogenology, Faculty of Veterinary Medicine, Assiut University, Assiut, Egypt; 2 Clinic for Obstetrics, Gynaecology and Andrology of Large and Small Animals with Veterinary Ambulance, Justus Liebig University, Giessen, Germany.

**Keywords:** dog, enzyme-linked immunofluorescence, immunochromatography, progesterone, radioimmunoassay

## Abstract

**Background and Aim:**

Measuring blood progesterone (P4) concentration has become an essential diagnostic tool in small animal reproductive medicine. Methods enabling precise and rapid on-site measurements are in high demand, especially for the optimization of breeding management in bitches. This study aimed to compare two commercial on-site methods (Speed™ P4, Virbac [M1] and mini VIDAS®, bioMérieux [M2]) and a well-established radioimmunoassay (RIA), which was used as a reference method.

**Materials and Methods:**

Comparative measurements were performed on 52 blood serum samples collected from 45 clinically healthy bitches of different breeds. The dogs had been presented to determine the estrus cycle stage and predict the time of ovulation. Each sample was divided into three aliquots. In aliquot 1, P4 was measured immediately applying M2. Aliquots 2 and 3 were stored at −20°C until analysis was performed using RIA and M1. The consistency of the three methods was investigated by pairwise linear regression analyses.

**Results:**

In RIA, the P4 concentrations ranged between 1.1 and 25.4 ng/mL. Regression analyses revealed highly significant (p<0.0001) positive correlations between the three methods applied (M1 vs. RIA: R=0.94; M2 vs. RIA: R=0.98; and M1 vs. M2: R=0.91).

**Conclusion:**

The results show that the two commercial on-site methods tested exhibit approximately equal, high consistency with the radioimmunological reference method and can, therefore, be used beneficially in a clinical setting. However, biological interpretation of data must be performed in a method-specific manner.

## Introduction

Progesterone (P4) measurement plays an essential role in modern small animal reproductive medicine, focusing specifically on dogs [1,2]. The primary source of P4 found in the systemic circulation of cyclic female domestic mammals is the luteal cells of the ovary. Elevated P4 concentrations may, therefore, qualitatively indicate the presence of endocrine-active luteal tissue. In bitches, luteinization of granulosa cells, which, as in other species, begins before ovulation, is accompanied by an increase in peripheral P4 concentrations that can be readily detected. Therefore, P4 measurement can be used in combination with clinical methods to optimize breeding/insemination management [3,4]. P4 measurement can also be used to detect the prepartum P4 decline and, thus predict the parturition time [5,6]. Other indications for P4 measurement in dogs are pregnancy monitoring, diagnosis of luteal insufficiency, and the detection of residual ovarian tissue in suspected cases of incomplete castration [7]. However, when interpreting the measured values, the often considerable individual variability must be considered, and, if necessary, method-specific reference values must be applied [3].

Several easy-to-perform rapid tests for determining P4 concentrations have been brought to market [8-10]. While in a purely scientific setting, the suitability of a method for measuring hormones depends primarily on its accuracy and precision, their usefulness in a clinical setting is determined by additional factors. In this context, it can be of great importance to perform the measurement on site, without extensive laboratory equipment and with an acceptable amount of work and time [8,9]. In bitches, the rapid availability of results from P4 determination is of particular importance in the immediate periovulatory phase, as the increase in P4 concentration accelerates considerably during this period [11,12]. The first methods for on-site measurement of P4 in bitches were semi-quantitative methods, in which the P4 concentration was estimated by comparing the color reaction with the provided color scale [9]. However, semi-quantitative methods are significantly less accurate compared to quantitative measurements. In addition, their usefulness in the application of cryopreserved semen is considerably reduced due to the upper limit of the measurement range generally being restricted to 5 ng/mL [3]. This is due to the fact that for the transfer of frozen semen, the early post-ovulatory period is chosen, in which P4 levels between approx. 10 and 20 ng/mL are measured. Therefore, in modern small animal reproduction, economically attractive methods that allow accurate and rapid on-site determination of P4 in canine blood over a wide concentration range are in high demand.

In 2011, Brugger *et al*. [13] demonstrated a commercial enzyme-linked immunofluorescence assay development to measure P4 in human blood running on an automated platform (mini VIDAS®, bioMérieux, France) allows the precise measurement of the hormone in canine blood samples. Subsequently, this method has been increasingly used in small animal practices and clinics. In 2017, an immunochromatographic method was launched for the quantitative measurement of P4 in canine blood samples (Speed™ P4, quantitative evaluation of the signal using the Speed™ Reader fluorescence scanning device, Virbac, France).

This study aimed to perform comparative measurements between the two commercial methods and a well-established radioimmunoassay (RIA), which was used as a reference method.

## Materials and Methods

### Ethical approval

This study was conducted in accordance with national laws and the methods approved by the Animal Welfare Committee of the competent regional authority (Regierungspraesidium) of Giessen, Germany, with the permission number V54-19c20 15h02Gl18/14kTV13/2017.

### Study period and location

The study was conducted from May 2019 to August 2019. The study was conducted in central Germany (Hessen).

### Donor animals and sample collection

Fifty-two blood samples from 45 clinically healthy bitches of different breeds aged 4.5±1.5 years were presented at the Clinic for Obstetrics, Gynaecology, and Andrology of Large and Small Animals, Justus Liebig University were used for this study. In seven animals, sampling was carried out twice on different days. One sample was collected during the first day of proestrus and one during the first day of estrus. The blood samples were collected from the antebrachial vein or the lateral saphenous vein and stored in sterile tubes (4 mL tube, 75×12 mm Z, Sarstedt, Nümbrecht, Germany) without the addition of an anticoagulant. The blood was allowed to coagulate for 15 min at 18-20°C (room temperature) and then centrifuged at 3000× *g* for 10 min (Rotina 35 R, Hettich Lab Technology, Tuttlingen, Germany). Each serum sample obtained was divided into three aliquots. Aliquot 1 was analyzed immediately with mini VIDAS^®^, while the aliquots 2 and 3 were stored at −20°C for a maximum of 2 months until P4 was measured using RIA or Speed™ P4 (tube 3.5 mL, 55×12 mm, Sarstedt, Germany).

### P4 determinations

P4 measurement using Speed™ P4 (Virbac, Bad Oldesloe, Germany) is based on the lateral flow immunochromatography technique. Sample preparation and subsequent P4 determinations were performed according to the manufacturer’s instructions. At the end of the incubation period, the signal was measured with the corresponding fluorescence scanning instrument Speed™ Reader (Virbac, Bad Oldesloe, Germany), which calculates the resulting P4 concentration. The measurement range of the Speed™ P4 method was between 1 and 20 ng/mL. The analysis took 15 min, starting from the moment the processed serum sample was applied to the test strip. The variability of the results was stated by the manufacturer to be approx. 10%.

P4 determination using mini VIDAS^®^ (bioMérieux, Nürtingen, Germany) is based on the competitive enzyme-linked fluorescence assay technique. Sample preparation and measurement were performed as instructed by the manufacturer. The measurement range extends between 0.25 and 80 ng/mL. In a previous study to validate this method with respect to its use in dogs, intra-assay coefficients of variation of 2.6-6.6% were reported in samples with concentrations of 6.8-51.4 ng/mL. Interassay variation coefficients ranged from 2.1% to 3.1% for samples between 15.1 and 49.1 ng/mL [14]. Performing the measurements on a batch of up to 12 samples takes approximately 45 min.

The RIA used as a reference method is essentially identical to the method of Hoffmann *et al*. [14] using tritium-labeled tracer and charcoal adsorption to separate free and antibody-bound steroids. Its use in canine blood plasma or serum has been established extensively [13,15-17]. The antiserum used was obtained after immunization of a rabbit against 4-Pregnen-11a-ol-3,20-dione hemisuccinate-bovine serum albumin (BSA) and exhibited the following cross-reactivity: P4: 100%, pregnenolone: 0.69%, 17a-OH-P4: 0.49%, testosterone: 0.37%, androstenedione, estradiol-17b, estrone, and cortisol: <0.01%. The standard curve included eight standards with P4 levels ranging from 40 to 2560 fmol/tube. Before radioimmunological determination, 0.1 mL of serum was extracted twice with hexane. The pooled extracts were dried in a vacuum evaporator (MicroDancer, Hettich AG, Baech, Switzerland) and redissolved in phosphate-buffered saline pH 7.2 containing 0.1% BSA. The intra- and inter-assay variation coefficients were 8.8 and 8.9%, respectively. All measurements were performed as duplicate determinations. Using a sample volume of 0.1 mL, the standard method covers a concentration range from 0.1 to 8 ng/mL. For concentrations above the standard curve, the measurement was repeated with a smaller sample volume.

### Statistical analysis

The consistency between the three tested methods was examined using pairwise linear regression analyses applying Microsoft® Office Excel 2007 (Microsoft Corporation Redmond, WA, USA).

## Results

**Figure-1:**
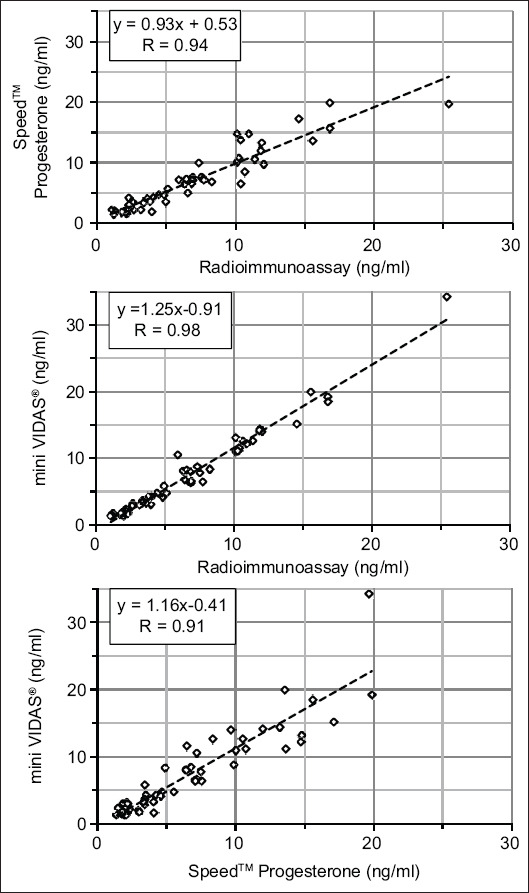
Pairwise comparison of tested progesterone measurement methods and results of correlation analyses (linear regression).

The results show that the two commercial on-site methods tested exhibit approximately equal, high consistency with the radioimmunological reference method (Figure-1). In each case, the pairwise linear regression analyses showed highly significant (p<0.0001) positive correlations and high positive correlation coefficients. The correlation coefficient (R) was highest for mini VIDAS® versus RIA (R=0.98) followed by Speed™ P4 versus RIA (R=0.94) and Speed™ P4 versus mini VIDAS^®^ (R=0.91). While mini VIDAS® significantly overestimated P4 concentrations at concentrations above 1 ng/mL compared to RIA (slope: 1.25, intercept: −0.91) and Speed™ P4 (slope: 1.16, intercept: −0.41), the regression line for Speed™ P4 versus RIA was close to the identity line (slope: 0.93, intercept: 0.53). With Speed™ P4, in one of the samples, a P4 concentration of 19.7 ng/mL was measured. The concentration measured in this sample was 25.4 ng/mL with RIA and 34.3 ng/mL with mini VIDAS (Figure-1), clearly indicating that the P4 concentration of this sample significantly exceeded the measurement range defined for Speed P4™. Omitting this sample from the linear regression analysis comparing Speed™ P4 to RIA resulted in a slope of 1.02, a Y-axis intercept of 0.03 for the regression line, and a correlation coefficient of R=0.94.

## Discussion

In the past decades, measurement methods for the analysis of steroid hormones were only available in a few specialized research laboratories. Nowadays, a wide range of corresponding commercial kits or services are available. However, the underlying methods have mainly been established and validated primarily for use in humans. The fact that the structure of steroids is species independent often leads to the uncritical application of measurement methods across species. Even when, as in the case of steroids, the structure of the analyte is species-independent, species-dependent matrix effects can cause a particular analysis method that works well in one species to have limited or no applicability in other species. The presence of cross-reacting molecules, binding proteins, or assay interference by lipid-like unidentified substances is considered possible reasons for matrix effects in steroid analysis. To overcome matrix effects, extraction of the samples (liquid-phase extraction or solid-phase extraction) may be applied or releasing reagents may be added to displace the analyte from unspecific binding sites. In any case, a method for measuring steroids must be validated specifically for the target species and sample matrix. This is especially true for methods without prior sample processing (direct assays), which usually include the commercially available methods for use in dogs [18,19].

The results confirm previous observations that mini VIDAS® enables the precise measurement of P4 in canine blood samples [13]. However, as shown by the slope of the regression line, this method significantly overestimates P4 concentrations compared to the applied RIA. Therefore, specific reference values should be used to interpret the results of the mini VIDAS^®^ method. However, it should be taken into account that the RIA used is an extraction assay in which approx. 5-10% of the analyte is lost due to preceding sample extraction. As an alternative to using method-specific reference values, the results obtained with mini VIDAS^®^ can be approximated to the RIA measured values by multiplying the data with a constant factor of 0.8. In a linear regression analysis “RIA” versus “transformed mini VIDAS^®^ data,” the regression line related to the samples used in this study results in y=0.97x+0.92. This confirms that multiplying the mini VIDAS^®^ data with the proportionality factor of 0.8 allows the use of the RIA reference values in dogs. The validity of this proportionality factor must be checked at regular intervals using comparative measurements. In the sense of “perfect analytics,” which demands not only acceptable precision but also correct results, a subsequent “mathematical correction” of the results may justify criticism. However, extensive experience clearly proves the benefit of this procedure in veterinary practice. Compared to the use of method-specific reference values, this procedure has considerable advantages. If, for example, the manufacturer was to make changes to a commercially available test system, it would not be necessary to establish new reference values, which usually involves a great deal of effort. Instead, the conversion method can be adapted based on a manageable number of comparative measurements. The literature describes such a case in which the manufacturing company altered a P4 measurement method established for use in humans and commonly used in dogs. While the modification did not affect the results in human sample material, the measured values were significantly lower in bitches after the modification [20].

As it is evident from the regression line, the Speed™ P4 method exhibits a high consistency with the RIA. This result is also in line with data from an earlier evaluation on canine blood samples. This method was compared to an electrochemiluminescence immunoassay (Elecsys, Roche Diagnostics GmbH, Mannheim) [21]. Thus, notwithstanding a slightly lower correlation coefficient than in the mini VIDAS® versus RIA comparison, the Speed™ P4 method can be considered a valuable and reliable method for on-site measurement of P4 in dogs. Somewhat disadvantageous is the upper limit of the measuring range to 20 ng/mL. However, accurate differentiation of values above 20 ng/mL is required only in a few clinical cases, such as monitoring P4 concentration during pregnancy [22]. As our observations presented above suggest, values slightly below 20 ng/mL should be interpreted with some caution, as the actual concentration may be significantly higher. In cases of concentrations around or above 20 ng/mL, retesting could be performed by appropriately diluting the sample material with “zero-serum” (e.g., serum from neutered dogs) to obtain more accurate readings.

## Conclusion

The present study was the first to scientifically evaluate the suitability of the Speed™ P4 rapid test for progesterone measurement in the bitch. Based on the results of this study, it can be concluded that this methodology can thus be used for the determination of mating time, just like the mini VIDAS, which has been available for some time. However, it must be noted that the verification was only carried out for the periovulatory period.

## Authors’ Contributions

AW and HAH: Designed the study. TC and HAH: Sample collection. GS: Analysis and interpretation of the data. GS and AW: Drafted the manuscript. All authors read and approved the final manuscript.
